# Space, time, and context drive anticipatory behavior: Considerations for understanding the behavior of animals in human care

**DOI:** 10.3389/fvets.2022.972217

**Published:** 2022-09-06

**Authors:** Bethany L. Krebs, Karli R. Chudeau, Caitlin L. Eschmann, Celina W. Tu, Eridia Pacheco, Jason V. Watters

**Affiliations:** ^1^Animal Wellness Department, San Francisco Zoological Society, San Francisco, CA, United States; ^2^Animal Science Department, University of California, Davis, CA, United States

**Keywords:** animal welfare, welfare indicator, reward sensitivity, zoo animal, welfare assessment

## Abstract

Animal-based measures reflecting the welfare state of individuals are critical for ensuring the well-being of animals under human care. Anticipatory behavior is one potential animal-based measure that has gained traction in recent years, as it is theorized to relate to animals' reward sensitivity. It is of particular interest as an assessment for animals living under human care, as the predictability of the captive environment lends itself to the development of this class of behaviors. Animals are likely to exhibit anticipation in locations related to the anticipated event, often in temporally predictable time frames, and before specific contexts they experience in their day-to-day management. In this sense and under certain circumstances, anticipatory behaviors are likely to drive observed behavioral or space use patterns of animals under human care. Drawing conclusions from such data without identifying anticipation may result in misleading conclusions. Here we discuss how space, time, and context are related to patterns of anticipatory behaviors in animals under human care, how unidentified anticipation may alter conclusions regarding animal behavior or welfare under certain circumstances.

## Introduction

Anticipatory behavior is a common phenomenon, documented in numerous species (1–9), in facilities where animals are cared for by humans (10). Briefly, anticipatory behavior is a suite of behaviors exhibited by animals during the appetitive phase (i.e., the searching phase of a behavioral sequence), aimed at the acquisition of a resource (11, 12). Readily observable anticipation is likely to develop under conditions where the availability of resources is predictable, either due to timing or cues in the environment (4, 13).

The past decade has seen a proliferation of animal welfare focused studies that assess anticipatory behavior to gain an understanding of animals' emotional states (14–17). This growth in research is evident in zoos and aquariums (hereafter zoos) where accreditation standards globally increasingly require assessing the welfare of all species living in zoological institutions (18–21). Anticipatory behavior provides a unique opportunity to study psychological states of animals and the factors influencing them with little manipulation. Indeed, in comparison to other approaches thought to assess animals' own reflections of their underlying psychological state, such as cognitive bias assessments, observations of anticipatory behavior rely on minimal intervention (9, 22, 23).

The manifestation of anticipatory behavior is greatly affected by many factors. Animals under human care generally receive primary reinforcing components of their care either on fixed schedules, following reliable or semi-reliable cues associated with them, or some combination of these. Thus, animals may anticipate based on the timing of events or the timing and sensory modality of the cues associated with these events (4, 24–26). The physical structure of the environment in which the behavior occurs can influence this behavior, as positive opportunities commonly occur in the same location(s) in the animals' space, such as feeding in a regular location or training sessions occurring where staff can access the animal (9, 23, 25). Additionally, the contexts in which the events occur can influence the manifestation of anticipatory behaviors (27, 28). Context itself can be multifaceted with variations in season, social situation, and cyclically hormonally-driven motivations (28–31).

Anticipatory behavior can be observed in animals that live in either captive or wild settings. Liberal interpretations of the behavior include all forms of behaviors associated with appetitive responses aimed at the acquisition of any perceived need (11, 32, 33). More conservative interpretations include responses tied to a clearly discernible (by the observer) cue(s) or to an observable pattern in the timing of events (11, 32, 33). Animals in wild settings thus express anticipatory behavior in a variety of ways and the general class of behavior is a core component of an ecologically relevant behavioral time budget.

Early studies labeled anticipatory behavior as ‘food anticipatory activity’ and demonstrated this behavior can become quite pronounced when animals rely on humans for scheduled caretaking (6, 13, 34, 35). These studies also occurred in laboratory settings where the factors that can shape the behavior were greatly simplified. Zoos provide more complex environments than those typically afforded lab animals, but are also subject to similar issues of scheduled care events, which can foster the development of anticipatory behavior (10). More recently, zoos have undergone a strong shift toward a focus on animal welfare (36) and emulating environments more in line with those the animals evolved in (37); nevertheless, much of the described behavior in zoo animals, the manner by which they utilize space, engage in daily rhythms, and even interact with conspecifics is shaped by patterns of anticipatory behavior. This may be particularly true in older descriptions that predate a focus on providing animals enriched environments (38) and longer lasting opportunities to be engaged in their environment (39). Anticipatory patterns across species appear to have specific relationships to the environmental contexts, timing of daily care events, and the spaces animals experience in their daily lives. Thus, unidentified anticipatory patterns have the potential to alter conclusions drawn from behavioral observations (40).

Here, we review how anticipation is expressed across space, time, and under different contexts. We discuss potential challenges of drawing conclusions from behavioral data collected from animals exhibiting anticipatory behaviors, and potential methods to identify or account for anticipation within existing datasets.

### Anticipatory behavior

Anticipatory behavior is a suite of behaviors, expressed by animals during the appetitive phase, or before a desired outcome is acquired (10, 33). This class of behaviors is goal-directed, and aimed at acquiring desired outcomes (10, 33). In this paper, we will use the phrase anticipatory behavior to refer to animals' responses toward positive outcomes such as: breeding opportunities, positive social interactions, or food, and also behavioral opportunities to obtain primary reinforcers such as positive reinforcement training or enrichments (8, 9, 25, 41). Animals can express anticipation toward negative or unpleasant events as well (42, 43). Given the focus of modern accredited zoos is on providing positive quality of life and minimizing pain or distress (19, 20, 44), for the purposes of this paper we will focus on anticipation of positive outcomes.

As a welfare indicator, anticipatory behavior is thought to indicate an animal's own perception of its reward sensitivity (3, 45–47). Animals in a positive state of well-being are expected to exhibit frequent but low intensity anticipatory behavior toward known rewards. Animals in a more negative state may show infrequent but intense anticipation toward known positive outcomes (10). In essence, animals with fewer positively reinforcing opportunities will intensely anticipate the rare events they do receive. Intense anticipation may appear similar to an abnormal repetitive behavior such as pacing (10, 40). With further consideration of the timing, context, and location of the behavior, it may be possible to distinguish between abnormal behaviors and anticipatory patterns (40). Anticipatory behavior itself is neither a positive nor negative welfare indicator, rather the intensity with which it is expressed has been suggested as a graded welfare indicator for individual animals (9, 10).

Anticipatory behavior is not one single behavior, but rather a suite of behaviors an animal expresses ahead of acquiring a predictable reward to prepare to engage with the opportunity (9), and can take several forms. The first is an increased level of activity ahead of gaining the desired outcome. A generalized increase in locomotion or activity has been documented across taxa ahead of predictable feedings (2, 25, 30, 31, 46–53). Alternatively, animals may sit and wait for the arrival of the anticipated outcome (23, 31, 54). Studies suggest differences between species in how anticipation is expressed (23, 31). Given anticipatory behavior is expressed across many species and is prone to developing under predictable conditions, it may be a complicating factor in interpreting behavioral data collected on animals living in human care. Behavioral data is often used to inform animal management decisions and draw conclusions about animal welfare (14, 55, 56). Understanding how anticipation influences animals' use of space, and varies with the timing and context of behavioral observations may thus have far reaching impacts on the care of captive animals.

### Anticipatory behavior and space use

Anticipatory behavior develops from the learned association between a temporal or other cue and an outcome (4, 24, 26). As animals learn to associate a time or stimuli with an event, they are also likely to learn the location the event happens as well. When the timing and location of a positive outcome are both unpredictable, evidence suggests animals vary their space use and behavior (57). This response may be related to how animals have evolved to express appetitive/anticipatory behaviors—in measured amounts throughout the day. In many zoos, caregivers or keepers provide opportunities in predictable places due to necessary constraints on exhibit access. In the same way animals can learn to associate unintentional cues provided by keeper presence with positive events (26), animals learn to associate specific places with predictable events occurring there (22, 58, 59). The learned associated between a desired event and a location may result in the development of anticipatory behaviors. The relationship between anticipatory behavior and space use will depend on which style of anticipation individuals express. For example, animals showing a sit-and-wait anticipatory pattern may approach an area they are fed, then sit or stand nearby until they are fed (23, 31, 54). The space use by this individual would not vary measurably during the anticipatory period. Animals exhibiting more active anticipation may repeatedly approach areas an event happens while stopping to look, listen, or otherwise gather information about whether the desired event is about to occur (25, 60, 61). Information gathering behaviors are also likely to be directed toward where the event is expected to occur, specifically if the event is dependent on caretaker presence (8, 9, 61). If there are several vantage points from which animals can gather information (e.g., about the location of their keepers), animals may move rapidly between two points while anticipating, pausing to listen or watch at each (60). An animal exhibiting this type of anticipation may show space use in a limited area of their enclosure, perhaps along an apparently fixed path. The active form of anticipation is potentially more likely to be (mis-)identified as an abnormal repetitive behavior.

Studies of animal space use in zoos have utilized a variety of methods (62), and have been used to draw conclusions about animal welfare (63–66), enclosure suitability for a species (67, 68), and species level preferences or needs for substrates (68, 69). A common assumption of many space use assessments is that varied space use is preferable to animals using only a limited portion of an enclosure (62). As an anticipating animal may only be using a small portion of its exhibit, space use data collected in the anticipatory period may indicate a lower diversity in space use measures. This may be particularly problematic for studies assessing enclosure suitability or substrate preferences for a given species.

For example, anticipating dolphins have been observed spending time at the surface of their pools, waiting and watching for their trainers' approach (25). This study was designed specifically to measure anticipatory behavior. To this end, the researchers conducted observations immediately before training sessions when the dolphins received food as a reinforcer. In this example, the event the animals are anticipating is predictable to them, and the animals can gain additional information about the arrival of the event by spending time in a specific area (i.e., the surface of the pool). If researchers collected data in the same time frame but did not know the training was about to occur, the observed space use and behavioral patterns may have been interpreted differently. If the data were used to assess pool depth preference, the conclusions may have suggested dolphins prefer using the surface rather than deeper parts of the pools. The lower activity levels and use of a smaller area could also be interpreted as signs of poor welfare in the time period before the training session. It should be noted, the same animals were observed after the training sessions and showed different behavioral patterns and fewer surface-oriented behaviors than during the anticipatory period (25).

### Identifying and accounting for anticipation in spatial data

Based on the previously described relationship between space and anticipation, several space use patterns may be of use in identifying anticipation. Clustered use of only a small area may indicate sit-and-wait form of anticipation occurring. Space use indicating a fixed path may be suggestive of the more active form of anticipation. Either form of anticipatory behavior would be expected to focus near where a desirable outcome is expected to occur. If the event is dependent on the presence of care staff, the animal's behavior may also be focused in areas where staff access the animal's enclosure.

Although we are discussing spatial data here, the distribution of data collection in time needs to be considered in determining whether anticipation might be influencing how animals use space. Balancing the start time of observations as much as possible throughout the day will help avoid undue influence of any specific management event. It is not uncommon for researchers in zoos to group data into broad pre-defined time periods (e.g., morning 10:00–12:00, afternoon 12:01–14:00, etc.,), depending on the animal's behavioral patterns or when it is most feasible to collect data. Data collection is then ideally balanced across all pre-defined time periods. This is a valid approach to addressing temporal variation in animal behavior. Within pre-defined time periods, however, the start times of observation sessions may not be balanced throughout the entire time period. For instance, perhaps due to timing constraints, ‘morning’ observations are started most days at 10:00, but the ‘morning’ time period extends through noon. If the animal receives its daily morning feed at 10:30 on most days, its behavior at 10:00 may differ from its behavior at 11:30. The behavioral observations throughout the entire day may be balanced between “morning” and “afternoon” time periods, while still overrepresenting an anticipatory period in the “morning”. Thus, the animal's space use between 10:00 and 10:30 may not be representative of how the animal uses its enclosure when it is not waiting to be fed. Ensuring there is some variation in the start time of observations within broader pre-defined time periods will keep anticipation from unduly influencing the observed patterns of animal space use.

If space use is being used to determine whether anticipation is occurring, examining the animal's space use throughout the day at the same shorter timescale will be useful to verify space use patterns suggestive of anticipation. If a particular time period shows evidence of anticipation, it may be beneficial to exclude these data from analyses related to space use. Analyzing how animals use space outside of anticipatory time periods may provide a more independent measure of how the animal interacts with its enclosure or substrates independent of management events.

### Anticipatory behavior and time

By definition, anticipatory behavior is dependent on time, as anticipation occurs before a predictable outcome (13, 24, 70, 71). Outcomes can become predictable to animals either by happening at approximately a similar time every day (53, 72, 73), being cued (intentionally or not, (8, 23, 26), or some combination of the two. Vertebrates have a well-developed internal clock, allowing them to develop a sense of when predictable events will occur in captive settings (24, 74). Reliable or semi-reliable cues animals learn in relation to caretaker behavior or environmental conditions can lead to anticipatory behavior as well (34, 75, 76). Feeding is commonly used to set, or entrain, circadian rhythms in laboratory studies (34, 77–79). The timing of feedings effectively set animals' internal clocks and circadian rhythms. Studies of rats and mice in laboratories have used wheel running as an index for activity level, and have quantified wheel revolutions throughout the day in relation to timing of feeds (80, 81). Measures of wheel running have provided insight into how anticipatory behaviors are expressed as predictable events approach. Specifically, anticipatory activity begins at low levels of intensity at time points before a predictable event, increases as the time of the expected event approaches, and then drops off suddenly when the desired event arrives. The sudden cessation of anticipatory behavior occurs when the animal is able to consummate the motivation the anticipation was directed toward (81). This structured temporal pattern of behavior can be contrasted with abnormal repetitive behaviors. Abnormal repetitive behaviors are typically described as functionless, and can result from varied etiologies (10, 82, 83). Based on the current understanding of these behaviors, there is no theory to suggest a temporal structure to when animals would express abnormal repetitive behaviors. Thus, this well-documented temporal pattern of behavior in anticipating animals shows the most promise as a diagnostically relevant factor for differentiating these classes of behaviors (40).

Food anticipatory activity has been documented in a wide variety of species (5, 8, 22, 23, 25, 27, 44, 48, 49, 56, 84, 85); however, the majority of this research was conducted in laboratory settings. Few studies outside of laboratories have examined how long before an event anticipation begins, nor what factors might impact the onset of anticipatory behaviors. Laboratory studies suggest food anticipatory activity tends to increase within an hour of expected feedings (13, 81, 82). Whether sit-and-wait anticipation is also expressed in a similar time frame is not known. Logistically, it may be more difficult to quantify changes to this style of anticipation over time. Increasingly rapid locomotion or location changes can be quantified, but measuring the intensity of an immobile behavior is challenging.

Animal behavior research has emphasized the value in understanding the relative importance of different resources to animals under human care (28, 84–86). As some resources will matter more than others depending on an animal's current state, animals can demonstrate behaviorally how much a given resource ‘matters’ to them by how much effort they will put in to obtain it (87, 88). Similarly, we may expect animals may express anticipation differently toward different resources. One study of domestic hens (*Gallus gallus*) demonstrated that the intensity of anticipation varies according to how much the reward is valued (89), and a study of a captive sea lion (*Zalophus californianus*) indicated the animal expressed more intense anticipation toward the first feed of the day compared to later feeds (9). As many non-domesticated species show seasonality of behavior and physiology associated with changes in behavioral drives, we may expect seasonal variations in anticipation as well. The extent to which an animal anticipates a particular event could vary seasonally, or the specific resources an animal anticipates may change throughout the year. For example, seasonal molting in birds increases the animal's energy requirements resulting in more food consumption (90, 91). Given the additional metabolic requirements of this process, animals may be more strongly motivated by food when they are undergoing a molt than at other times of the year. They may also exhibit more intense anticipation toward feedings than other opportunities during this time.

### Identifying and accounting for anticipation in temporal data

Statistical methods already used to account for variation over the course of the day or study period, or to account for temporal autocorrelation may be useful for accounting for variation in behavior over time due to anticipation. Specifically, using generalized linear mixed models with a random effect for time of day to analyze behavioral data may help account for periods of significant anticipatory behavior in a data set, or account for variation in sampling through time (92). Assessing the response variables for temporal autocorrelation and including a variance structure accounting for this may also help account for temporal patterns within the data (92). As generalized linear mixed model methods can also tolerate uneven sampling across time periods, somewhat unbalanced timing of observations can be accounted for using this modeling method. Accounting for seasonal or annual variation is often done utilizing this method in other fields, and this may be useful for longer term zoo research as well (92, 93).

Ensuring observations are generally balanced throughout the day is another practical way to account for temporal variation in behavioral patterns. Even if timing of observations is grouped into pre-defined time periods, ensuring observation start times within each time period are varied can help balance out any anticipation captured in the observations. As previously stated, descriptions of how long before an event anticipation might be expected to begin are lacking outside of laboratory studies. As such, assessing behavioral data at a relatively short temporal scale, such as hour by hour, for signs of anticipation may be advisable. If a specific time period shows a much higher or lower activity level, determining whether any management events of particular importance to the animal occur around that time may help identify the behavior as anticipatory. When possible, determining whether the animal shows an increase followed by a sudden decrease in a particular behavior (e.g., walking or pacing) may be definitively used to identify a pattern as anticipatory. This approach would require repeated behavioral observations at a fine temporal scale, and may not always be feasible. Depending on the behavioral variables of interest for the study, excluding anticipatory periods from further analysis may be warranted.

As anticipation is directed toward a specific outcome, it is important to understand not only the temporal patterns of the behavior but also what management events happen and when they typically occur in a given day. As accredited zoos focus more on ensuring good welfare for animals in their care, most animals receive multiple daily positive opportunities in the form of feeding, enrichment, positive reinforcement training sessions, changing social groups, and other management decisions aimed at providing a varied and stimulating environment (20, 94, 95). Zoo animals may anticipate any of these events, but anticipation is most likely to develop for events that occur repeatedly, around approximately the same time, and/or are preceded by a cue or string of cues (9, 26, 76). Understanding the general time frame of daily management events an animal receives will therefore be a critical piece of information for understanding when the animal may be expressing anticipation.

Finally, if a concern is raised regarding a behavioral pattern that appears to be abnormal, the temporal patterns of the behavior may be useful in distinguishing between abnormal repetitive behaviors and intense anticipation. Specifically, if the behavior in question increases over a short period of time, and then decreases rapidly or stops after the arrival of a management event, there would be reason to conclude the behavior is anticipatory in nature. If it is not feasible to conduct detailed behavioral assessments in the time period the behavior is generally observed, an interview with care staff regarding the animal's regular daily schedule may help establish a timeline for when rewarding events occur for the animal.

### Context

The factors we are referring to as ‘contexts’; in this review are any additional covariates that may impact study outcomes. Contexts or circumstances change in zoos throughout the day, weeks, or even months. As previously discussed, time and space are important and influence anticipatory behavior. For this paper, we are defining contexts as circumstances in a zoo that are out of the animal's control, and vary within space and time - essentially any covariate that can influence behavior. This is not a comprehensive list of all contexts animals experience in zoos, however we've attempted to broadly classify previous studies of relevant contexts here.

### Anticipatory behavior and contexts

The impact of many specific contexts on anticipatory behavior have not yet been explicitly explored. In general, the direction of the relationship between a given context and anticipatory patterns will depend on the animal's level of reward sensitivity (10, 11, 33). The relationship between context and anticipation will also depend on whether the individual perceives the context as a positive or negative outcome (5, 23, 42, 96). We are including context as a separate factor from space and time, because although contexts vary within space and time, anticipation also varies between contexts (61, 97–99). In turn, variation in context will influence how anticipation is expressed in time and space. For instance, an animal with varied enrichment may demonstrate its lower reward sensitivity through less intense anticipation (45, 100).

### Human contexts

One context that receives significant attention in all zoological institutions is the effect of humans. Visitors and care staff are present on a daily basis. Repeated interactions with humans may be considered human-animal relationships (HAR), and the relationships between animal care staff and the animals they care for can have implications for animal behavior and well-being (101–105). Studies have shown that HARs can have a positive, negative, or neutral effect for animals and depend on the quality and quantity of interactions between two individuals (105). A case study of two zoo animals suggests that animals under human care can find social interactions with non-caretaking humans positive, even when the interaction resulted in no primary reinforcement (23). This study demonstrated this social interaction was rewarding enough to lead to the development of anticipatory behaviors when the interaction followed a reliable signal (23). Besides the quality of an animal's relationship with its caretakers, keeper presence is one of the major factors that influences daily conditions animals experience (106). Keeper presence is often associated with positive events for the animal, and animals are generally highly attuned to cues related to their keepers (26, 105, 107). The arrival or presence of caretakers likely shapes daily patterns of animal behavior. The majority of an animal's feedings, enrichments, or training sessions will occur within a short time of a keepers' arrival (49, 108). For instance, dolphins anticipating positive reinforcement training sessions orient themselves according to keeper presence and activity (25). The context of care staff presence may therefore influence the timing and spatial components of animals' behaviors. Thus, an individual animal's experience of its relationship with its caretakers and the frequency of keeper visits both have potential to impact anticipatory patterns.

Zoo visitor presence is known to impact animal behavior in various ways. Interactions between visitors and zoo animals are a subset of human-animal relationships studied in zoos known as the visitor effect. The effect of visitor presence on animals is well–documented (109, 110). The nature of the impact that visitors have on animal behavior varies. Studies have shown varying levels of negative impact associated with high visitor density, including increased corticoid concentrations (111, 112), increased hiding behavior (113), increased abnormal repetitive behaviors (114, 115), and increased intra-group aggression (116). The impact of crowd size is variable, however, with some studies finding a negative relationship and others finding no impact, even in the same species (109, 117). Animals' response to visitor presence is likely influenced by species and individual personality (117, 118). To date, little or no research we could find has been done investigating the relationship between visitor numbers and anticipation in zoo settings. This may be an avenue for future investigation. How an animal's anticipatory patterns change with visitor numbers is likely to depend on whether it perceives visitor presence as aversive or enriching. The predicted relationship between reward sensitivity and intensity of anticipation can be useful in predicting how animals' anticipation may vary with visitor numbers (10). Animals finding visitor presence stressful would be expected to exhibit more intense anticipation under high visitor numbers. Animals experiencing visitor presence as enriching may exhibit minimal anticipation when visitor numbers are high. The potential for correlation between higher visitor numbers and events the animals perceive as high value may complicate such a study. Specifically, if trainings or feedings are advertised to zoo visitors, the timing of increased visitor numbers at the animal's exhibit and the time leading up to the management event may be confounded.

### Social contexts

The social context of animals also impacts many aspects of how they interact with their environments (see (119) for an in-depth review). The social context of an animal includes intra-specific interactions with conspecifics. The nature of intra-specific interactions is expected to vary with the size and composition of the group (120), as well as the individual temperaments of the group members (120, 121). An animal's social context may also include any individuals of another species with which the animal shares space (122, 123). Social context does not only include animals with physical access to each other, as both conspecifics or heterospecifics within the perceptible range of an individual animal may impact its behavior. For example, okapi (*Okapia johnstoni*) with visual access to conspecifics exhibit more pacing (124), and the sex-ratio of animals in surrounding pens impacts breeding behavior in giant pandas (125). In a mixed-species example, alarm calling and vigilance in brown capuchins (*Cebus apella*) decreased with the addition of a visual barrier between the primates and a small felid in a nearby exhibit (126). Studies of anticipation and social contexts in zoo animals are limited; however, laboratory studies indicate social interactions can have significant impacts on anticipatory patterns of individuals (46).

### Environmental contexts

It has long been recognized that inappropriate environmental conditions can compromise animal well-being. Due to this, zoo scientists are increasingly interested in empirically assessing the environmental conditions animals experience to ensure animals can achieve positive well-being. Assessments have examined animals' responses to the myriad environmental conditions they are subject to, such as artificial lighting (127, 128), sound levels (129–131), or the thermal environment (132, 133). Such environmental measurements may be the main focus of the study, or included as a covariate expected to impact animal responses (134, 135). Interest in the impact of complex changes to normal environmental conditions is also increasing, with more research being conducted on events held at or impacting zoos (136–139).

The most common method to provide changes to the environment is environmental enrichment. Environmental enrichment is a component of animal husbandry that aims to provide a dynamic environment through varied behavioral opportunities for animals under human care (62, 140–144). Environmental enrichment can take many forms, including feeding strategies, sensory, social, structural, and cognitive enrichments (143, 145). Giving enrichment daily is common, but the type of enrichment, frequency, and timing can vary between enclosures, species, and zoos. Type, frequency (times throughout the day), timing, and location of enrichment can be an essential context to consider when collecting behavioral data. Studies in farm animals indicate a variety of animals exhibit anticipatory behavior ahead of receiving environmental enrichment opportunities (5, 30, 144). Enriched environments are generally associated with indicators of positive well-being in animals, such as increased engagement with their environments (146–148), positive judgment biases (149, 150), and play behaviors (41, 151, 152). Enrichments providing problem solving opportunities have also been associated with lowered intensity of anticipatory behaviors (60), as well as other indicators of positive well-being in animals (153, 154).

### Identifying and accounting for anticipatory behavior in relation to contexts

The contexts an animal experiences are likely to interact with anticipation by modulating the animal's overall reward sensitivity (10). As already stated, any outcomes the animal finds to be positive are candidates for the animal to express anticipation toward, and the more of these an individual experiences the less intense overall anticipation is expected to be. Thus, when a study aims to alter one or more contexts an animal experiences, gaining as complete a picture of what the individual's ‘normal’ day comprises ahead of any changes is critical. This is already a common feature of many studies in zoos, with baseline data collected ahead of any manipulations to the environment or animal management. Alongside the collection of baseline behavioral data, understanding the timing, frequency, and individual preferences for various contexts and events study animals experience in their daily lives can provide a more complete understanding any resulting changes observed during the study.

Context is also included here as it is expected to vary in both space and time, suggesting animals may be experiencing their environments differently throughout the course of the day. This seemingly basic statement has important implications for anticipatory patterns of individual animals. It is common for zoo animals to be shifted into publicly visible spaces when the zoo opens, and they receive a portion of their daily diet and novel enrichment for the day. By later in the day, the enrichment has been engaged with or emptied of food, and the animal's diet may be consumed. The environment the same animal experiences 4 h after shifting may be significantly different in terms of context than the environment it shifted into in the morning, with potentially fewer behavioral opportunities available (26, 39). Thus, the biological relevance of the animal's environment is likely to change throughout the day. The timing of events in relation to one another and potentially the order of events may all be important contexts to consider as well.

Anticipation may be an unrecognized source of variations among behaviors of group-living individuals, as each individual has the potential to experience a given context differently. For example, a more dominant group member may have the opportunity to exploit feedings or enrichments first, or subordinate individuals may not receive as many positive social interactions with other group members. Less dominant animals may thus be expected to display more intense anticipation on average than more dominant individuals. Ruling out whether this is the case may help account for results when a change is observed in behavior at the group level; but, the outcome is driven by a single animal's response. Considerations of context will necessarily vary according to what the overall question of a study is.

## Conclusion

Throughout this review, we discussed space, time, and context separately—but in practice, all of these factors are interconnected. How animals use space or experience different contexts are constantly changing through time. Understanding space use, temporal patterns, or contexts influencing animal behaviors requires concurrent understanding of each of the other factors in many cases.

We have identified several specific patterns of how anticipatory behaviors are expressed in relation to space, time and contexts, based on reviewing the existing research. Specifically, if a behavior is anticipatory, it would be expected to (1) occur in an area proximal to where a positive event occurs (2) increase in frequency or intensity as the time of a predictable positive outcome approaches (3) cease to be expressed when consummation of the motivation occurs and (4) be modulated by other contexts expected to change individual's reward sensitivity (e.g., decrease in intensity with increased opportunities to obtain rewards and vice versa). These patterns can be useful for identifying anticipation in animals living under human care.

The extensive body of research into how animals use their spaces, respond to changes over time, and other contexts influencing animal behavior have been a major part of the zoo animal welfare field. As the focus of animal management and care moves toward the goal of providing more choice, control, and complexity for animals, the methods used for measuring how animals respond to these changes need to shift as well. By integrating spatial and temporal considerations explicitly into how we measure animal behavior, we can improve our understanding of the prevalence of anticipatory behaviors, and clarify how these behaviors may have inadvertently shaped our conclusions about animals' preferences and requirements.

## Data availability statement

The original contributions presented in the study are included in the article/supplementary material, further inquiries can be directed to the corresponding author.

## Author contributions

BK conceptualized the manuscript. BK, KC, CE, CT, and EP wrote the manuscript. BK, KC, CE, CT, EP, and JW provided significant edits to the manuscript. JW is the principle investigator and provided logistical support to all contributors. All authors contributed to the article and approved the submitted version.

## Conflict of interest

The authors declare that the research was conducted in the absence of any commercial or financial relationships that could be construed as a potential conflict of interest.

## Publisher's note

All claims expressed in this article are solely those of the authors and do not necessarily represent those of their affiliated organizations, or those of the publisher, the editors and the reviewers. Any product that may be evaluated in this article, or claim that may be made by its manufacturer, is not guaranteed or endorsed by the publisher.
